# Hydatidform Mole (Myxoma Chorii), with Report of Case

**Published:** 1908-09

**Authors:** J. B. Cranner

**Affiliations:** Wilmington, N. C.


### HYDATIDIFORM MOLE (MYXOMA CHORII), WITH REPORT OF CASE.

BY J. B. CRANMER, M. D., WILMINGTON, N. C.

In writing this paper, it has not been my purpose to touch upon the histology of the chorion, nor to dwell at length upon the pathology of the Hydatidiform Mole.

I do not claim to be giving you an original treatise; this is largely a resume of the subject, with the report of an interesting case.

The degeneration, (aside from the normal process of atrophy) that may affect the chorionic villi, is of two kinds—cystic and fibro-myxomatous. This paper has to do with the first—cystic degeneration of the chorionic villi.

This pathological condition is characterized by hypertrophy of the chorionic villi, and by their conversion into cysts, varying in size from that of a millet seed, to that of a grape, or even to that of a hen's egg, connected with one another and with the base of the chroin, by pedicles of varying breadth. The ovum grows rapidly, with consequent expansion of the uterus. There is escape of blood from the uterine cavity into the vagina, and a premature expulsion of the ovum, which is covered with small, transparent cysts. An embryo may or may not be found.

There has been much discussion of this condition, by reason of the mystery which formerly surrounded its origin. Even at so early a date as the sixth (6th) century, papers with no very clear idea of its nature, were written upon this subject. De-Graaf held that the vesicles were *mature ova,* while some authors thought that each one represented an early pregnancy.

It is probable that many of the extraordinary cases of multiple gestation recorded in the early literature, were instances of the Hydatidiform Mole, as, for example, that of the Countess of Hagenau, who was believed to have given birth to three hundred and sixty-five (365) embryo at a single labour.

Priestly goes so far as to quote, (in reference to this case), from a writer of that time: "that the Countess Margaret brought forth at one time, three hundred and sixty-five infants—one hundred and eighty-two males, one hundred and eighty-two females, and the odd one an hermaphrodite." This as late as 1276 of our era.

Pepys even records in his diary that he visited the house in which this remarkable delivery occurred, and saw the *brass platters* upon which, according to a custom of the day, the children were carried before the Bishop of the diocese, for baptism.

Numerous theories have been advanced as to the nature of the lesion, until Virchow, in 1853, stated, as his belief, that the process was essentially a myxomatous degeneration of the connective tissue of the chorion villi, and designated it as Myxoma Chorii.

Marchand, however, in 1895, held that the essential feature of the affection was to be found, not so much in the stroma, as in the epithelial covering the villi.

The process occurs at a time when the villi are almost equally developed over the whole ovum, before the third month, and involvement of the whole villi is the rule. Sometimes, the placenta alone is affected. In this morbid process, it is important to know, that the cells of Langhan's layer and the syncitium display an exuberant growth, showing a decided inclination to penetrate uterine tissue; therefore, the relation of myxoma of the chorion to syncitial cancers is quite intimate, and in a large proportion of the latter growths, there is associated a cystic disease of the chorion villi. There may be metastasis of the whole chorion villi without malignant degeneration of the epithelial cells, or the chorion epithelium may undergo malignant degeneration after metastasis.

Aside from the possibility of the development of a deciduoma malignum, the hydatidiform mole is a serious affection.

Dorland, for example, noted a mortality of 10 per cent. with the one hundred instances which he collected from literature.

As I said before, the prominent symptoms associated with cystic degeneration of the chorion, are:

*First.*—A rapid increase in the size of the uterus.

*Second.*—A discharge of blood, or blody serum, and,

*Third.*—An escape of vesicles.

This last symptom is of rare occurrence, and the first is not always typical, so that the clinical phenomena in the case of vesicular mole, do not always admit of a definite diagnosis.

None of us care to make a practice of reporting single cases, but, as few of us have an opportunity to see many *like cases,* we may be pardoned for making occasional deductions from our individual experiences.

A case which came to me a few months ago, has awakened my interest on this subject. Mrs. W., age twenty, (20); family history, negative; personal history showed previous vigorous constitution. Married ten months; menstrual period having been missed for five months. Slight hemorrhage, pain and discharge having occurred once during this period, at which time, patient was kept in bed by her attending physician, under usual treatment, for prevention of miscarriage. August 30, 1907, I was called for the first time, at twelve o'clock, mid-night, to what was reported to me over the 'phone as a "miscarriage case." Upon arriving at the house, I was told by a woman present that " it was all over." I found the patient flowing profusely, having already expelled a quantity of grape-like masses. To be sure that uterine cavity was entirely emptied, I made pressure above, and another large mass of like character was expelled. The hemorrhage was continuing to an alarming extent. Giving hypodermically 20 minims of ergotole, one-thirtieth (1-30) of a grain of strychnine, and one-eighth (1-8) of a grain of morphine sulphate, I made careful digital examination, as soon as it was practicable. The uterus was found much enlarged, flabby, and the cervix patulous. Small particles of the grape-like masses were found adherent to the uterine walls. With lightest possible touch, and dullest rinsing curette, I went over the walls of the uterus, and washed out the cavity with a weak antiseptic solution. The hemorrhage was almost immediately controlled.

The patient made a good recovery, and is now in perfect health, although still very much impressed by the information I gave her as to the rarity of such cases, seeming rather proud of being, according to Madam Boivin, a case in twenty thousand.

